# Effects of Stacking Configuration on Impact Resistance of Electric Locomotive Coupling Protective Covers Reinforced by CFRP, GFRP, and Their Hybrids

**DOI:** 10.3390/ma18133133

**Published:** 2025-07-02

**Authors:** Yanhui Xu, Jiyong Chen, Mingzhu Guan, Shoune Xiao, Guangwu Yang, Dongdong Chen

**Affiliations:** 1 Technical Center, CRRC Datong Co., Ltd., Datong 037038, China; xuyanhui_3@163.com (Y.X.); chenjiyong1367@126.com (J.C.); 2 State Key Laboratory of Rail Transit Vehicle System, Southwest Jiaotong University, Chengdu 610031, China; snxiao@swjtu.cn (S.X.); gwyang@home.swjtu.edu.cn (G.Y.);

**Keywords:** fiber-reinforced composite materials, interlayer hybridization, coupling protective cover, structural impact, ballast impact

## Abstract

In the context of global environmental pollution and energy shortages, the use of lightweight designs of railway vehicles has become a key technological approach to improve energy efficiency and reduce carbon emissions. The use of lightweight and high-strength materials such as carbon-fiber-reinforced composites to replace traditional metal vehicle structures holds great application potential. In this study, random track loads and ballast impact loads that may occur during service were considered, and a finite-element model of the electric locomotive coupling protective cover was established. The impact resistance of CFRP, GFRP, and their interlayer hybrid configurations (C/G/C and G/C/G) against structural and ballast impacts were investigated. The calculation results showed that the CFRP protective cover exhibited the best structural impact resistance (with the lowest Tsai–Wu strength failure values), but it also had the largest maximum deformation displacement (2.36 mm) under ballast impact conditions. In contrast, the GFRP protective cover had a higher Tsai–Wu strength failure value, indicating that it had worse structural impact resistance, but it had a lower maximum deformation displacement (2.20 mm) under ballast impact conditions, demonstrating superior ballast impact resistance. The impact resistances of the hybrid-layered protective covers fell between those of the CFRP and GFRP in terms of the structural impact, while their ballast-impact resistance surpassed those of single-fiber configurations.

## 1. Introduction

Carbon-fiber-reinforced polymer (CFRP) possesses advantages such as a high specific stiffness/strength, excellent fatigue resistance, and strong design flexibility [1,2,3,4]. It has garnered significant attention in lightweight structural design across various fields, including aerospace, aviation, railway vehicles, and automobiles [5,6,7,8,9]. In the case of railway vehicles, the typical applications of CFRP have gradually transitioned from non-load-bearing structures, such as cabin decorations, to primary load-bearing structures, including driver cabins, equipment compartments, car bodies, and skirts [10,11,12]. During service, railway vehicle structures are frequently subjected to various types of impact loads, such as structural impact loads from vehicle acceleration/braking and impact loads caused by foreign objects like ballast or hail [13,14,15,16]. Due to the invisibility and complexity of CFRP structural impact failures, extensive research and development efforts have been made globally for a long time [17,18,19,20,21].

A substantial amount of research has been devoted to investigating the impact-response behaviors of composite structures. Cascino et al. [22] designed a lightweight roof structure for a light rail vehicle using CFRP, achieving a 63% weight reduction compared to that of the original design while maintaining structural-impact performance. Graupner et al. [23] studied the cyclic loading fatigue behavior of flax/glass hybrid materials used in railway wheel axles and found that with an increasing number of load cycles, flax-based fiber-reinforced polymer exhibited a better fatigue performance than GFRP. The fatigue strength of the flax/glass hybrid material was between those of the two materials, while it also exhibited superior bending and impact properties compared to those of pure-flax-based composites. Chen et al. [24] experimentally and numerically investigated the axial energy-absorption behaviors of CFRP, Glass-fiber-reinforced polymer (GFRP), and their interlayer hybrid structures. They found that under a 45° chamfer-triggered mode, CFRP tubes exhibited the best crashworthiness, whereas under the same 45° chamfer and embedded trigger mechanism, GFRP tubes exhibited superior energy-absorption characteristics compared to CFRP, with an average crushing force that was 98.5% higher. Tian et al. [25] conducted impact-response studies on CFRP energy-absorbing structures at the ends of railway vehicles using trolley impact tests and finite-element simulations. They examined chamfer-triggered (TI) and embedded-triggered (TII) modes and found that both trigger modes led to progressive failure behaviors at the ends of the energy-absorbing tubes. In the TI mode, the average crushing force was 891.89 kN, and the specific energy absorption was 38.69 kJ/kg. In comparison, the TII mode resulted in reductions of 21.2% and 21.9% in the average crushing force and specific energy absorption, respectively. Zhu et al. [26] fabricated lattice-core sandwich panels based on cosine function unit cells using aluminum alloy and CFRP as face sheet materials. Low-velocity impact tests were performed to study the impact damage mechanisms of the structure. The study revealed that the aluminum-alloy face sheet sandwich panels had better impact resistance than CFRP face sheet panels. Furthermore, increasing the cell size or reducing the cosine-period length effectively decreased the indentation depth.

In the study of hybrid composite materials for resistance to foreign-object impact, Hazell and Appleby [27] investigated the ballistic impact responses of carbon/aramid hybrid laminates. They found that the optimal impact resistance was achieved when CFRP was used as the front-facing impact layer and Kevlar fibers as the backing layer. Grujicic et al. [28] conducted a simulation study on the ballistic impact response of carbon/Kevlar hybrid armor. They discovered that while the stacking sequence had a limited effect on the impact resistance when the laminate thickness was fixed, it significantly influenced the projectile trajectory. Ma et al. [29] compared the impact responses of unidirectional, woven, and unidirectional/woven hybrid laminates under the impact energies of 10, 17, and 25 J. Their findings revealed that unidirectional laminates exhibited the most severe matrix cracking and delamination, while woven laminates had the smallest delamination area but suffered from significant fiber fracture failure. Meanwhile, the unidirectional/woven hybrid laminate exhibited the least severity in matrix cracking, delamination, and fiber breakage. Liu et al. [30] examined the effect of the aramid fiber volume fraction on the impact responses of aramid/carbon fiber hybrid laminates. They found that as the aramid fiber content increased, the specific energy absorption (SEA) of the hybrid composite first increased and then decreased. The failure mode transitioned from brittle fracture to interlayer delamination and interface debonding, and the composite maintained high structural integrity and an excellent post-impact residual performance. Wu et al. [31] experimentally studied the effect of the interlayer hybridization of CFRP and Kevlar on the high-speed impact resistance of laminates. They developed a simulation model that considered the asymmetric tensile/compressive elasto-plastic behavior of Kevlar. Their study revealed that incorporating Kevlar fibers altered the failure mode of the laminate from the brittle fracture of carbon fibers to fiber pullout from the Kevlar fiber bundle, significantly improving the impact resistance of the CFRP laminates.

Studies have shown that fiber hybridization has enormous potential for enhancing structural-impact resistance and decreasing component weight [32,33,34,35]. However, most current studies on structural-impact resistance in railway vehicles have focused on metal structures, with very few on composite components. Only a few researchers [36,37,38,39] have conducted localized impact-response studies on composite structures using experimental and numerical-simulation methods. Furthermore, these studies have primarily focused on single-material composites such as CFRP or GFRP under simplistic loading conditions, without evaluating the applicability of their findings to different operating conditions. In railway vehicle component design, however, multiple working conditions must be considered. With this background, the coupling protective cover of an electric locomotive was investigated in this study. In Section 2, finite-element models for structural impact and ballast impact on the protective cover are established. Implicit analysis (ANSYS) and explicit analysis (ABAQUS) were employed for structural and impact simulations, respectively, considering four stacking sequences: CFRP, G/C/G, C/G/C, and GFRP. Section 3 presents the effects of different stacking configurations on the structural-impact and ballast-impact performance of the protective cover. The present study fills significant research gaps in impact-resistant protective structures and hybrid fiber-composite optimization, while providing a dedicated solution for rail transit vehicles.

## 2. Finite-Element Model Development

### 2.1. Geometric Model and Layering Design

**Figure 1:**
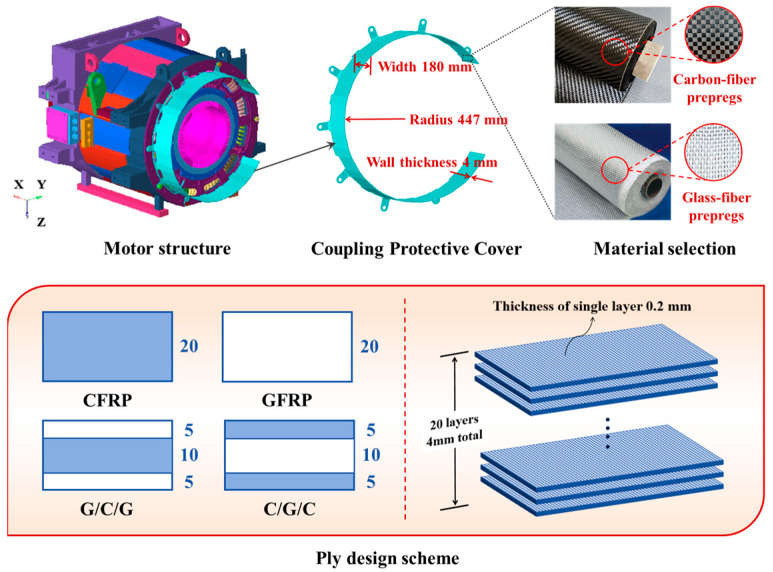
Coupling protective cover solid model and stacking design scheme.

The finite-element model consisted of a motor structure comprising a coupling protective cover and the main motor body, as shown in Figure 1. During vehicle operation, the protective cover must withstand structural-impact loads caused by vehicle vibrations while also shielding the internal motor structure from splashing ballast, sand, and other debris. The coupling protective cover in the model had a wall thickness of 4 mm, a radius of 447 mm, and a width of 180 mm, and it was attached to the motor body using M20 × 60 bolts. The coordinate system was defined as X for the transverse direction, Y for the longitudinal direction, and Z for the vertical direction.

Compared to unidirectional composite materials, two-dimensional woven composites exhibit more balanced mechanical properties and better delamination resistance. Considering the impact-protection requirements of the coupling protective cover, four fiber layup designs were proposed using plain-woven CFRP and GFRP as raw materials: CFRP, G/C/G, C/G/C, and GFRP, as illustrated in Figure 1. All layup schemes followed a [0°/90°]20 configuration, with a single-layer fabric thickness of 0.2 mm and a total thickness of 4 mm. The G/C/G configuration consisted of five layers of GFRP symmetrically placed on the outer surfaces with a core of ten layers of CFRP, while the C/G/C configuration had five layers of CFRP on the outer surfaces and ten layers of GFRP in the core. Throughout this study, the material coordinate system is defined with X and Y representing the in-plane orthogonal directions aligned with the 0° (warp) and 90° (weft) fiber orientations of the woven fabric, respectively, and Z denoting the out-of-plane thickness direction. The mechanical properties used in the calculations were obtained through mechanical testing and are listed in Table 1.

### 2.2. Structural Impact Finite-Element Model

**Figure 2:**
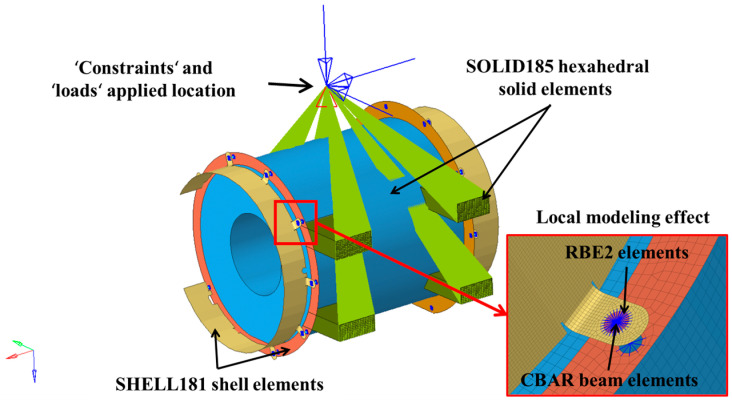
Finite-element model of structural-impact condition.

A structural impact finite-element model of the composite coupling protective cover was developed using the general-purpose finite-element software HyperMesh 2017. The protective cover was discretized using SHELL181 shell elements with an element size of 3 mm. The simplified motor-body structure was discretized using SOLID185 hexahedral solid elements with an element size of 10 mm. The green wedges represent the simplified geometric profile of the motor housing, providing essential structural boundary conditions to ensure accurate modeling of the motor’s operational environment. CBAR beam elements were used to simulate the bolts, which were connected to the components using RBE2 elements. The model consisted of 768,115 nodes and 735,005 elements, including 647,380 hexahedral elements, 87,569 shell elements, one mass element, 18 beam elements, and 37 RBE2 elements. The overall discretization of the motor is shown in Figure 2.

To simulate the excitation caused by track irregularities during rail-vehicle operation, constraints were applied at the bolt-hole positions of the motor-suspension bracket, which connected to the bogie, and full-field accelerations were applied in three directions at the constrained points. According to the impact conditions specified in the GB/T 21563-2018 standard [40] for bogie-suspended motors, six impact conditions, which included gravitational acceleration, were formulated for the protective-cover structure, as shown in Table 2. The boundary conditions are illustrated in Figure 2. The structural impact strength of the coupling protective cover was calculated using the Ansys solver.

The three-dimensional Tsai–Wu failure criterion was employed to validate the computational results for each impact condition. This criterion accounts for the interactions between the three-dimensional stress components and the differences between the tensile and compressive strengths of the material, as expressed by the following equation [41]:

(1) $$\left\{\begin{array}{c} A=-\frac{{\left(\sigma_{x}\right)}^{2}}{X_{T}X_{C}}-\frac{{\left(\sigma_{y}\right)}^{2}}{Y_{T}Y_{C}}-\frac{{\left(\sigma_{z}\right)}^{2}}{Z_{T}Z_{C}}+\frac{{\left(\sigma_{xy}\right)}^{2}}{{\left(S_{xy}\right)}^{2}}+\frac{{\left(\sigma_{yz}\right)}^{2}}{{\left(S_{yz}\right)}^{2}}+\frac{{\left(\sigma_{xz}\right)}^{2}}{{\left(S_{xz}\right)}^{2}}\\ +\frac{C_{xy}\sigma_{x}\sigma_{y}}{\sqrt{X_{T}X_{C}Y_{T}Y_{C}}}+\frac{C_{yz}\sigma_{y}\sigma_{z}}{\sqrt{Y_{T}Y_{C}Z_{T}Z_{C}}}+\frac{C_{xz}\sigma_{x}\sigma_{z}}{\sqrt{X_{T}X_{C}Z_{T}Z_{C}}}\\ B=\left(\frac{1}{X_{T}}+\frac{1}{X_{C}}\right)\sigma_{x}+\left(\frac{1}{Y_{T}}+\frac{1}{Y_{C}}\right)\sigma_{y}+\left(\frac{1}{Z_{T}}+\frac{1}{Z_{C}}\right)\sigma_{z}\\ \xi =A+B \end{array}\right.$$

where *ξ* represents the three-dimensional Tsai–Wu failure criterion value; *σ_x_*, *σ_y_*, *σ_z_*, *σ_xy_*, *σ_yz_*, and *σ_xz_* (MPa) are the six stress components at any given point; and *C_xy_*, *C_yz_*, and *C_xz_* are the Tsai–Wu coupling coefficients, generally with values of −1. If the Tsai–Wu failure criterion value *ξ* is less than 1, the material remains in the linear elastic state; if it exceeds 1, the material has failed.

The Tsai–Wu failure criterion was implemented in the Ansys solver to evaluate the failure indices of single-fiber/interlayer hybrid composite protective covers, with the material properties of carbon-fiber and glass-fiber composites listed in Table 3 serving as inputs for the failure-criterion calculation.

### 2.3. Gravel Impact Finite-Element Model

**Figure 3:**
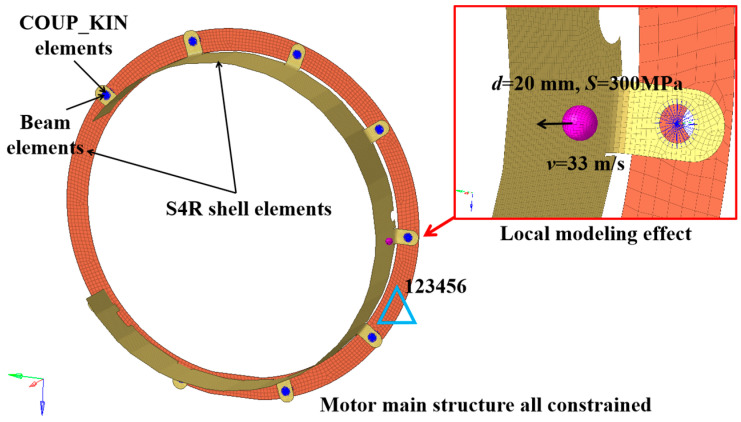
Finite-element model of ballast-impact condition.

The process of the ballast impact on the protective cover was simulated using the ABAQUS/Explicit 2021 software. The protective cover was meshed with S4R shell elements, with an element size of 3 mm. To improve the computational efficiency and simulation accuracy, the connected components of the motor body were also discretized using S4R shell elements with an element size of 10 mm. The bolts were simulated using Beam elements and were connected to the components using COUP_KIN elements. Boundary conditions were established by constraining the six degrees of freedom at the bolt connection parts between the coupling protective cover and the motor body, as shown in Figure 3.

Table 4 presents the impact conditions for gravel collision with the protective cover, where the number of ballast stones was one. The impact velocity of the stone was determined by referencing the actual operating speed of the railway vehicle (maximum 120 km/h), resulting in a calculated impact speed of 33 m/s. The impact direction was aligned with the normal vector of the shell elements, representing the most severe impact condition. The stone was simplified as a sphere with a diameter of 20 mm and a uniaxial compressive strength of 300 MPa. This value was determined by comprehensively considering the mechanical properties of potential impact objects encountered during train operation. The ballast model was simulated using C3D8R hexahedral elements, with an element size of 4 mm.

The built-in VUMAT subroutine in ABAQUS, combined with a failure criterion based on continuum damage mechanics theory, was used to simulate the intralaminar progressive damage of the laminated fabric. By inputting the material parameters, the constitutive model could be directly invoked. The two-fiber fabric-reinforced composite materials used in this study were both orthotropic. The constitutive model, named “ABQ_PLY_FABRIC_,” defines the in-plane constitutive relationship as follows [42]:

(2) $$\left\{\begin{array}{c} \varepsilon_{11}\\ \varepsilon_{22}\\ \varepsilon_{12}^{el} \end{array}\right\}=\left[\begin{array}{ccc} \frac{1}{(1-d_{1})E_{1}} & -\frac{v_{12}}{E_{1}} & 0\\ -\frac{v_{21}}{E_{2}} & \frac{1}{(1-d_{2})E_{2}} & 0\\ 0 & 0 & \frac{1}{(1-d_{12})2G_{12}} \end{array}\right]\left\{\begin{array}{c} \sigma_{11}\\ \sigma_{22}\\ \sigma_{12} \end{array}\right\}$$

where *ε* is the elastic strain vector, *σ* is the stress vector, *E*_1_ and *E*_2_ are the Young’s moduli in the principal in-plane material directions (circumferential direction and axial direction, respectively), *G*_12_ is the shear modulus, *v*_12_ and *v*_21_ are the principal Poisson’s ratios, *d*_1_ and *d_2_* are fiber-fracture failure parameters along the in-plane principal directions, and *d*_12_ is the failure control parameter in the in-plane shear direction. In the elastic loading phase, *d*_1_, *d*_2_, and *d*_12_ are all equal to 0. If the applied stress exceeds the material strength in a given direction, the corresponding damage parameter becomes greater than 0. The fracture energy per unit area under tension/compression along the fiber direction is 80 kJ/m^2^ for both loading modes.

### 2.4. Model Verification

**Figure 4:**
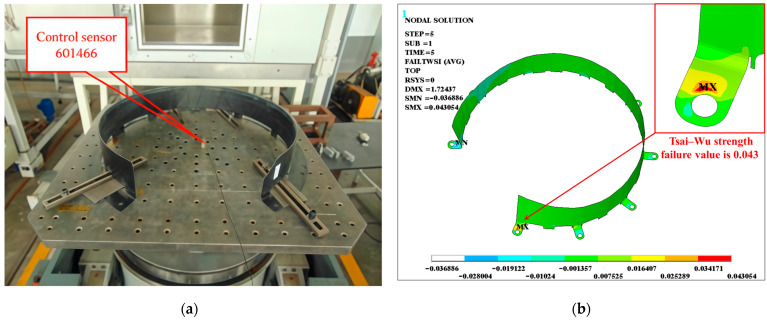
Comparison of test and simulation results. (**a**) The experiment site; (**b**) simulation result of Condition 5.

To validate the numerical model of the CFRP protective cover, specimens were fabricated using a vacuum-assisted molding process and were subjected to structural impact tests in accordance with the GB/T 21563-2018 standard, under conditions identical to those specified in Condition 5 (refer to Table 2). As shown in Figure 4a, the experimental results indicate that the specimens exhibited no visible damage post-impact. Subsequent numerical simulations were conducted to analyze the impact response under the same conditions. The simulation results (Figure 4b) revealed that the maximum Tsai–Wu strength failure values occurred in the bolt hole connection area; however, this value remained below one, indicating that the structure did not reach the failure threshold and retained a safety margin. The excellent correlation between experimental observations and simulation outcomes confirms the reliability of the established model in predicting the impact response of CFRP protective covers.

## 3. Results Analysis

### 3.1. Structural-Impact Conditions

**Figure 5:**
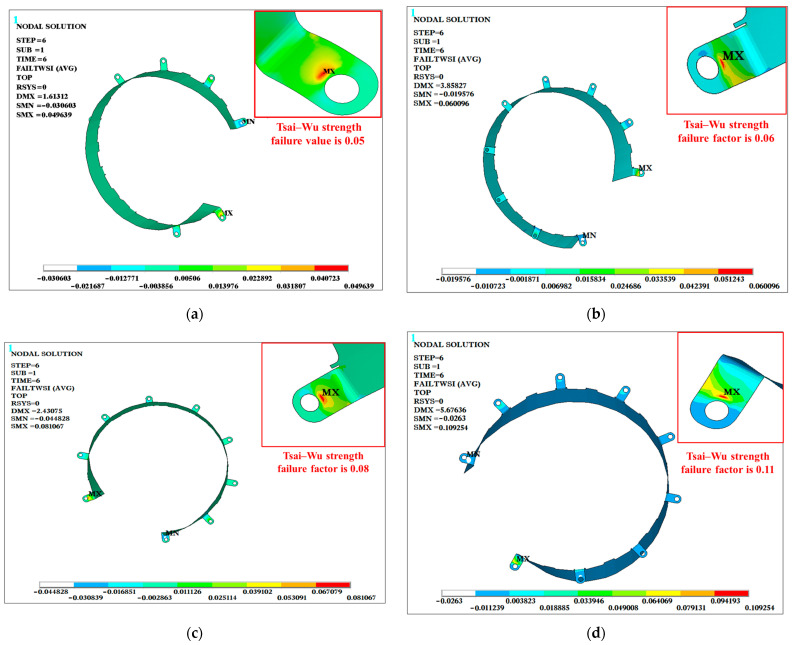
Tsai–Wu strength index contour plots for composite protective covers. (**a**) CFRP; (**b**) G/C/G; (**c**) C/G/C; (**d**) GFRP.

Table 5 summarizes the simulated results of the maximum Tsai–Wu strength failure values and their corresponding locations for the coupling protective cover under six structural-impact conditions for the four different stacking configurations (CFRP, G/C/G, C/G/C, and GFRP). It can be observed that the Tsai–Wu strength index values for all of the stacking configurations were less than one, indicating a certain safety margin. Condition 6 represents the most severe structural-impact condition, and Figure 5 illustrates the Tsai–Wu index contour plots for the four stacking configurations under this condition. The maximum Tsai–Wu coefficient for all of the protective covers was located at the bolt connection area between the protective cover and the motor, which was likely due to stress concentration caused by gradual structural shape changes in this region. The maximum Tsai–Wu failure factor for the CFRP structure was 0.05, whereas the GFRP configuration had a maximum Tsai–Wu failure factor of 0.11, indicating that GFRP had the weakest structural-impact resistance. The maximum Tsai–Wu failure factors for the C/G/C and G/C/G configurations fell between those of the CFRP and GFRP configurations.

**Figure 6:**
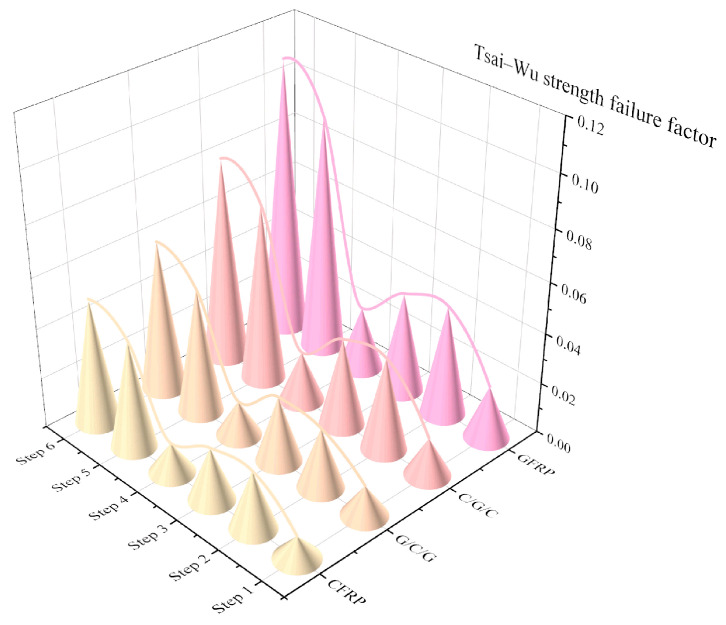
Comparison of Tsai–Wu strength failure factors for composite protective covers (steps 1–6 correspond to Conditions 1–6 in Table 2).

Figure 6 shows a three-dimensional bar chart illustrating the relationship between the stacking configurations, structural impact conditions, and maximum Tsai–Wu failure factors. The failure curves for the four protective cover configurations exhibited almost identical trends across the six conditions, demonstrating the good consistency of the finite-element simulation results. The most severe condition for material failure was the vertical −31 g impact (Condition 6), while the least severe conditions were the longitudinal −30 g impact and the vertical −1 g impact (Condition 4). Across all conditions, the Tsai–Wu strength failure values for the CFRP, G/C/G, C/G/C, and GFRP stacking configurations showed an increasing trend with a higher GFRP content, indicating that introducing GFRP led to a deterioration in structural performance. The maximum Tsai–Wu failure factor of the G/C/G configuration was lower than that of the C/G/C configuration, suggesting that the G/C/G configuration provided better structural-impact resistance.

### 3.2. Ballast Impact Conditions

**Figure 7:**
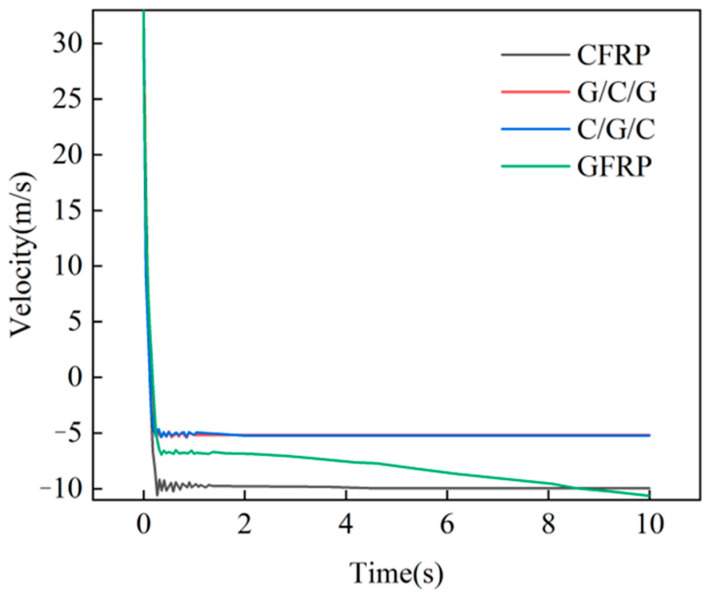
Velocity–time curves of ballast stone during impact.

Figure 7 presents the velocity–time curve of the ballast along the impact direction (*Y*-axis) during the protective-cover impact process. It shows that after the stone block contacted the protective cover, its velocity decreased linearly and eventually reached a stable state. Moreover, the final velocities of all of the material configurations were less than 0, indicating that none of the protective covers were penetrated and the stone bounced back. The final velocities of the stone for the CFRP and GFRP protective covers were −10 m/s, which was higher than the value of −5 m/s for the hybrid material protective covers, suggesting that the hybrid material protective covers absorbed more energy during impact.

**Figure 8:**
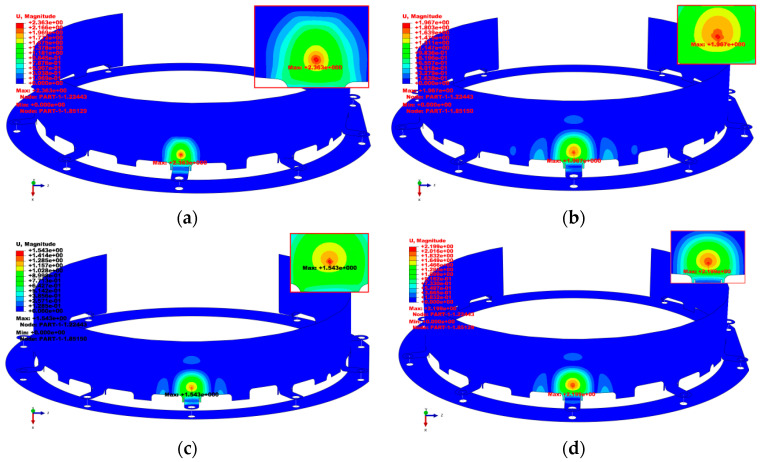
Deformation displacement contour plots of impact regions of composite protective covers. (**a**) CFRP; (**b**) G/C/G; (**c**) C/G/C; (**d**) GFRP.

Figure 8 shows the deformation contour plots of the impact region for all stacking configurations. The maximum deformation displacement of the CFRP protective cover was 2.36 mm, and that of the GFRP configuration was 2.20 mm, with the CFRP exhibiting a smaller deformation area. This may be attributed to the higher stiffness of the carbon-fiber material. The maximum deformation displacement for the C/G/C configuration was 1.54 mm, while that of the G/C/G configuration was 1.97 mm, corresponding to reductions of 34.75% and 16.53%, respectively, compared to that of the CFRP configuration. When compared to the GFRP configuration, these reductions were 30% and 10.45%, respectively. This indicated that after hybridizing CFRP and GFRP, the maximum deformation displacements of the structures were lower than those of pure carbon-fiber and pure glass-fiber configurations. Additionally, compared to the G/C/G configuration, the C/G/C configuration, which used carbon fiber as the outer layer, exhibited smaller deformation displacement. This was likely because the carbon-fiber layer absorbed most of the impact energy during the damage-propagation stage. This phenomenon is similar to the findings of Chen et al. [43], who investigated the low-velocity impact resistance of carbon/glass/basalt hybrid laminates. Their experimental results similarly demonstrate that the hybrid composite exhibits significantly better impact resistance than single-fiber systems, owing to the synergistic mechanisms of energy dissipation by basalt fibers, stiffness retention from carbon fibers, and damage delocalization via glass fibers.

**Figure 9:**
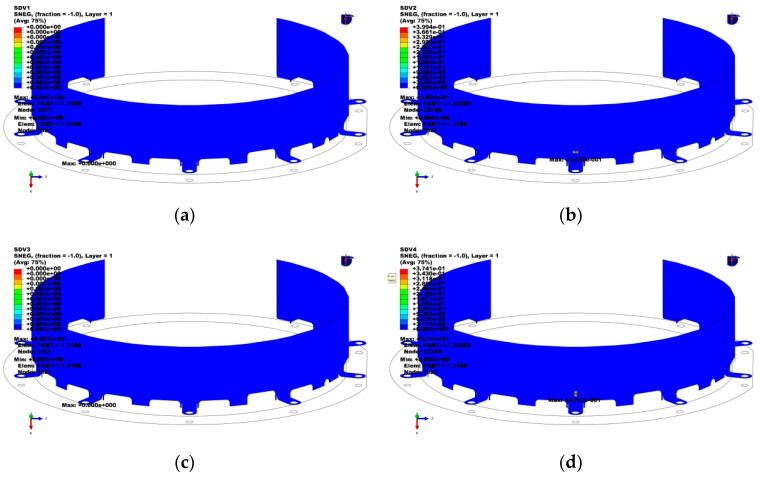
Material failure distributions of the C/G/C protective covers. (**a**) SDV1; (**b**) SDV2; (**c**) SDV3; (**d**) SDV4.

Figure 9 shows the Solution Dependent Variables (SDV) 1–5 material failure distribution plots for the C/G/C hybrid stacking configuration protective cover. SDV 1 and SDV 2 represent the tensile and compressive damage factors (d1) in the 0° (circumferential) direction, respectively, while SDV 3 and SDV 4 represent the tensile and compressive damage factors (d2) in the 90° (axial) direction, respectively. None of these damage factors exceeded 0.5, indicating that no penetration occurred during the impact process.

### 3.3. Discussion

**Figure 10:**
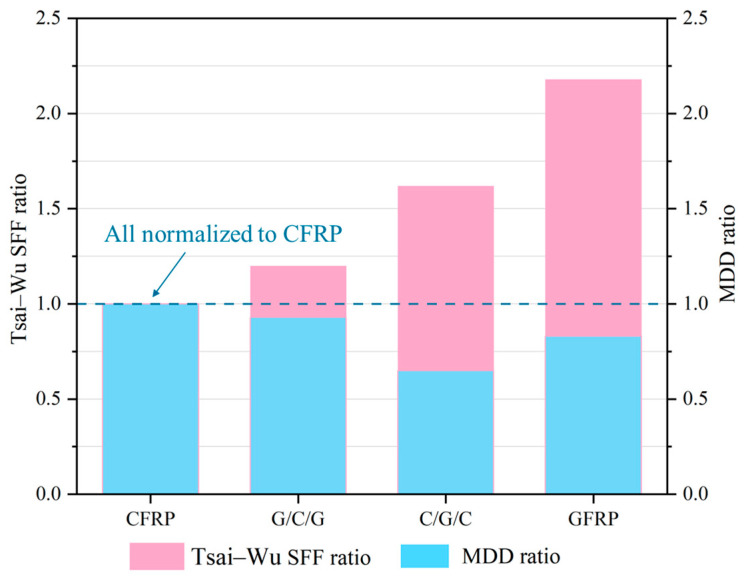
Comparison of the performance of protective covers with different stacking configurations.

Figure 10 presents a comparison of the structural-impact and ballast-impact performances for the four different stacking configurations of the protective cover. To facilitate a comparison, all results were normalized with respect to the CFRP results. As the GFRP content increased, the Tsai–Wu strength failure factor (Tsai–Wu SFF) ratio exhibited a gradual upward trend, whereas the maximum deformation displacement (MDD) ratio under ballast-impact conditions remained below 1.0. This phenomenon indicated that incorporating GFRP into the CFRP protective cover structure enhanced its resistance to ballast impact but reduced its overall structural-impact resistance. The Tsai–Wu SFF ratio of the G/C/G configuration was lower than that of the C/G/C configuration, indicating that the G/C/G configuration provided better structural-impact resistance. However, the MDD ratio of the G/C/G configuration was higher than that of the C/G/C configuration, suggesting that its ballast-impact resistance was weaker. The above analysis demonstrates that the optimal stacking configuration of the protective cover structure varied depending on the loading conditions. In actual structural design, it is necessary to comprehensively consider the specific loading conditions, stacking configurations, and structural safety requirements.

## 4. Conclusions and Perspectives

This study focused on the composite coupling protective cover of an electric locomotive. A simulation study on the impact resistance of CFRP, GFRP, and their interlayer hybrid stacking configurations under structural impact and low-velocity ballast impact conditions was conducted. The conclusions are as follows:

(1) Under structural impact conditions, the maximum Tsai–Wu coefficient values for all stacking configurations were concentrated at the bolt connection between the protective cover and the motor body but remained below one, indicating that the protective-cover structure met the safety requirements. The CFRP configuration had the smallest maximum Tsai–Wu coefficient, corresponding to the highest structural safety, whereas the GFRP configuration had the highest Tsai–Wu coefficient, making it the most vulnerable structural design. The hybrid stacking configurations fell between these two extremes;
(2) Under ballast-impact conditions, none of the protective covers were penetrated, confirming that they met the requirement for safe operation. The maximum deformation displacement of the CFRP protective cover was close to that of the GFRP configuration, but its deformation area was smaller. The maximum deformation displacements for the C/G/C and G/C/G hybrid stacking configurations were 1.54 and 1.97 mm, respectively, which were lower than those of both the CFRP and GFRP configurations. This indicated that hybridizing CFRP and GFRP improved the impact resistance of the structure;
(3) A comprehensive comparison of the simulation results under two operating conditions showed that incorporating GFRP into the CFRP protective-cover structure enhanced its ballast-impact resistance but reduced its overall structural-impact resistance. Furthermore, in the hybrid material design, using CFRP as the outer layer helped improve the structure’s ballast-impact resistance (with the lowest maximum impact deformation) but this led to the deterioration of the structural-impact resistance.

Through rational material selection and structural design, coupling protective covers with excellent impact resistance can be fabricated. Future research should focus on (1) establishing a multi-objective optimization model for carbon/glass fiber hybrid composites to systematically evaluate the cost-weight-performance trade-offs under industrial manufacturing constraints; (2) enhancing material performance through optimized manufacturing processes; and (3) designing accelerated lifetime tests according to EN 12663 standards [44] using rail-specific load spectra to investigate damage evolution under 10^7^ impact cycles, thereby validating the material’s durability under extreme operating conditions (e.g., high-frequency impacts) for more accurate prediction and assessment of impact resistance in hybrid composite coupling protective covers.

## Figures and Tables

**Table 1:** Composite material properties.

Material	Density (kg/m^3^)	Elastic Modulus (GPa)	Poisson’s Ratio	Strength (MPa)
CFRP	1400	EX: 75.6 EY: 75.6 EZ: 9 GXY: 4.82	0.05	Tensile strength: 800 Compressive strength: 500
GFRP	2600	EX: 24 EY: 24 EZ: 9 GXY: 4.82	0.1	Tensile strength: 414 Compressive strength: 331

**Table 2:** Structural-impact conditions.

Condition	Acceleration		
	Transverse X	Longitude Y	Vertical Z
1	+30 g	-	−1 g
2	−30 g	-	−1 g
3	-	+30 g	−1 g
4	-	−30 g	−1 g
5	-	-	+(30 − 1) g
6	-	-	−(30 + 1) g

**Table 3:** Material parameters for the failure-criterion calculation of composite materials.

Material Parameter	CFRP	GFRP
Stress in Tension	X: 800 MPa	X: 414 MPa
	Y: 800 MPa	Y: 414 MPa
	Z: 54 MPa	Z: 54 MPa
Stress in Compression	X: −500 MPa	X: −331 MPa
	Y: −500 MPa	Y: −331 MPa
	Z: −240 MPa	Z: −240 MPa
Stress in Shear	XY: 60 MPa	XY: 60 MPa
	YZ:60 MPa	YZ:60 MPa
	XZ: 60 MPa	XZ: 60 MPa
Stress Coupling Coefficients	XY: −1	XY: −1
	YZ: −1	YZ: −1
	XZ: −1	XZ: −1

**Table 4:** Ballast-impact conditions.

Condition	Ballast-Stone Diameter (mm)	Ballast-Stone Strength (MPa)	Impact Velocity (m/s)
1	20	300	33

**Table 5:** Maximum Tsai–Wu failure values and locations for four stacking configurations under different structural-impact conditions.

Condition	CFRP		G/C/G		C/G/C		GFRP	
	Tsai–Wu Strength Failure Value	Failure Location	Tsai–Wu Strength Failure Value	Failure Location	Tsai–Wu Strength Failure Value	Failure Location	Tsai–Wu Strength Failure Value	Failure Location
1	0.01	Bolt hole	0.01	Bolt hole	0.02	Bolt hole	0.02	Bolt hole
2	0.02	Bolt hole	0.02	Bolt hole	0.04	Bolt hole	0.04	Bolt hole
3	0.02	Bolt hole	0.03	Bolt hole	0.04	Bolt hole	0.04	Bolt hole
4	0.01	Bolt hole	0.01	Bolt hole	0.02	Bolt hole	0.03	Bolt hole
5	0.04	Bolt hole	0.05	Bolt hole	0.07	Bolt hole	0.09	Bolt hole
6	0.05	Bolt hole	0.06	Bolt hole	0.08	Bolt hole	0.11	Bolt hole

## Data Availability

The original contributions presented in the study are included in the article; further inquiries can be directed to the corresponding author.
